# A Highly Selective Hemicyanine-Based Turn-Off Fluorescent Sensor for Cyanide Detection in Food Samples

**DOI:** 10.1007/s10895-026-04788-3

**Published:** 2026-05-08

**Authors:** Fuat Gokbel, Ziya Aydin, Şeyma Akın, Esma Nur Çenet, Mustafa Keles

**Affiliations:** 1 Faculty of Engineering, Department of Food Engineering, Karamanoğlu Mehmetbey University, Karaman, 70200 Türkiye; 2 Vocational School of Technical Sciences, Karamanoğlu Mehmetbey University, Karaman, 70200 Türkiye; 3 Faculty of Engineering, Department of Bioengineering, Karamanoğlu Mehmetbey University, Karaman, 70200 Türkiye; 4 Faculty of Arts and Sciences, Department of Chemistry, Osmaniye Korkut Ata University, Osmaniye, 80000 Türkiye

**Keywords:** Chemosensor, Cyanide, Fluorescence, Hemicyanine, Nucleophilic addition

## Abstract

Cyanide is a highly toxic anion. Humans are commonly exposed to cyanide through the consumption of drinking water or by eating plants that contain cyanogenic glucosides, such as cassava, a key carbohydrate source in many diets. While numerous methods exist for cyanide detection, few are both cost-effective and simple enough for use by individuals without specialized training. In this work, we designed and synthesized a hemicyanine-based fluorescent sensor ZM-FES for the detection of cyanide (CN^−^). Among the anions tested, the sensor showed high selectivity towards CN^−^. The addition of CN^−^ to ZM-FES in aqueous solution (ACN/H_2_O (1:1, v/v)) caused a dramatic decrease in the absorbance and fluorescence intensities. The detection mechanism was based on nucleophilic addition between CN^−^ and the indolium group, which was confirmed by ^1^H NMR and mass spectral analysis. The fluorescence intensity plot as a function of CN^−^ concentration showed a good linear relationship in the range 0–10 µM, and the detection limit was calculated as 0.195 µM. Furthermore, the proposed detection approach can operate over a wide pH range from 2.1 to 9.3. Finally, ZM-FES was successfully utilized to detect CN^−^ in food samples and satisfactory results were obtained.

**Graphical Abstract:**

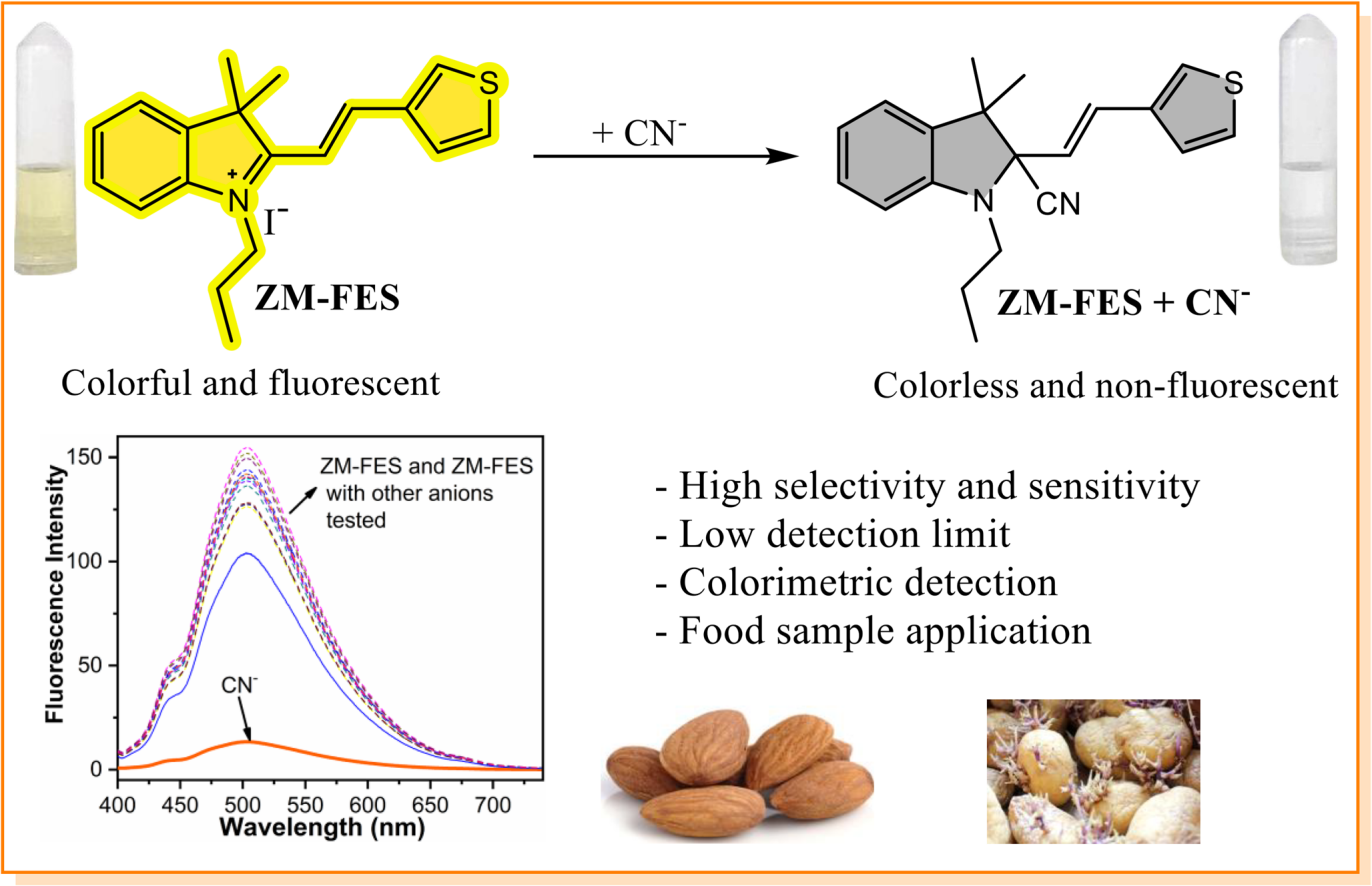

**Supplementary Information:**

The online version contains supplementary material available at 10.1007/s10895-026-04788-3.

## Highlights

- A hemicyanine-based turn-off fluorescent sensor (ZM-FES) for CN^-^ detection.
- ZM-FES showed excellent selectivity for CN^-^ ions.
- Low LOD for CN^-^ ions (0.195 µM) and well below the WHO acceptable limit for CN^-^ ions.
- The sensor was successfully applied to food samples.

**Supplementary Information:**

The online version contains supplementary material available at 10.1007/s10895-026-04788-3.

## Introduction

Anions play an important role in a wide variety of fields, including medicine, biology, catalysis and environmental science [1]. Among anions, cyanide (CN^−^) stands out due to its widespread use in industrial processes such as herbicides, electroplating, fiber production, petrochemical production, and gold extraction [2–4]. However, cyanide is also recognized as a highly toxic substance in both environmental and biological contexts [5, 6]. Cyanide ions interfere with vital biological processes such as cellular respiration and oxidative metabolism by binding to Fe³^+^ in metalloenzymes [7]. Fatal consequences can result from even minimal exposures of more than 0.5 mg per kilogram of body weight [8]. Therefore, the World Health Organization (WHO) has set the maximum allowable concentration of cyanide in drinking water at 1.9 µM to reduce the serious toxicity of cyanide [9]. It is essential to develop effective methods for monitoring and detecting cyanide anions.

Various strategies have been employed for cyanide detection, including mass spectrometry [10], electrochemistry [11], flow injection [12], ion chromatography [13], and colorimetric and fluorescent sensors [14–24]. Among them, fluorescent sensors have attracted great interest due to their numerous advantages such as versatility, low cost, ease of operation, portability, rapid response, and high sensitivity. The cyanide (CN^−^) fluorescent sensors reported to date are primarily based on nucleophilic addition [25], metal complex displacement [26, 27] and nanoparticles [28, 29]. Nucleophilic reactions involving oxazines [30, 31], acridinium salts [32], dicyano-vinyl groups [33–35], boronic acids [36, 37], aldehydes [38, 39], and indolium groups [23, 40–42] generally demonstrate satisfactory selectivity, owing to the strong nucleophilicity of CN^−^. Among these, indolium has recently emerged as one of the most promising reactive groups for CN^−^ detection, due to its positively charged nature, which generates a strong attractive interaction with CN^−^ [41]. However, many of these methods still face challenges in aqueous environments, such as a narrow pH working range and low sensitivity, which limit their applicability for real sample analysis.

In this study, the determination of CN^−^ ions was achieved using a fluorescent sensor based on nucleophilic addition. The sensor, ZM-FES, was synthesized through a two-step reaction involving the coupling of an indole moiety with a thiophene moiety. The optical and colorimetric characteristics of ZM-FES were assessed using naked-eye observation, UV-Vis and fluorescence spectrophotometry in ACN/H_2_O (1:1, v/v). ZM-FES displayed a rapid and highly selective response to CN^−^ ions, effectively distinguishing them from other anions tested. The interaction between ZM-FES and CN^−^ was further analyzed through mass and ¹H NMR titration experiments. For practical use, ZM-FES was applied to natural food samples, such as bitter almonds and sprouting potatoes. Trace amounts of CN^−^ ions in these samples were detected by the sensor.

## Experimental

### Materials and Instruments

All reagents, chemicals, and solvents utilized in this study were procured from commercial suppliers (Sigma and Merck) and were used as received without any additional purification. ¹³C NMR and ¹H NMR spectra were acquired using a Bruker Ultrashield Plus Biospin Avance III 400 MHz NaNoBay FT-NMR spectrometer at a temperature of 298 K. UV-Vis absorption spectra and fluorescence emission spectra were measured using a Shimadzu UV-1800 spectrophotometer and a Hitachi F-7100 fluorescence spectrophotometer, respectively. Mass analysis was conducted on a Bruker microflex LT MALDI-TOF MS spectrometer. The pH measurements were performed with an Ohaus-Starter 2100 pH meter.

#### Synthesis of 3,3-dimethyl-1-propyl-2-(2-(thiophen-3-yl)vinyl)-3 H-indol-1-ium (ZM-FES)

The compound 2,3,3-trimethyl-1-propyl-3 H-indole-1-ium iodide was synthesized following a procedure described in the literature [43].

2,3,3-Trimethyl-1-propyl-3 H-indol-1-ium iodide (0.330 g, 1.0 mmol) and 3-thiophenecarboxaldehyde (0.112 g, 1.0 mmol) were dissolved in 2 mL of ethanol. The mixture was stirred at 100 °C for 1 h. When the substances were completely dissolved, 20 µL of piperidine was added and stirred at reflux condenser at 100 °C for 16 h (checked by TLC). It was cooled to room temperature and the precipitated substance was filtered. It was washed with cold ethanol and dried in a vacuum oven. Light brown crystalline powder ZM-FES was obtained (0.2855 g, yield 67%). ^1^H NMR (400 MHz, CDCl_3_) δ 8.89 (s, 1H), 8.51 (d, *J* = 16.0 Hz, 1H), 8.13 (d, *J* = 6.2 Hz, 1H), 7.68 (d, *J* = 16.0 Hz, 1H), 7.63–7.59 (m, 2 H), 7.57 (s, 2 H), 7.45–7.41 (m, 1H), 4.94 (t, *J* = 7.2 Hz, 2 H), 2.05 (dd, *J* = 14.6, 7.3 Hz, 2 H), 1.90 (s, 6 H), 1.11 (t, *J* = 7.4 Hz, 3 H). ^13^C NMR (101 MHz, CDCl_3_) δ 182.73, 148.56, 143.52, 140.67, 138.61, 138.26, 129.70, 128.06, 127.57, 122.88, 114.74, 112.20, 52.60, 49.74, 27.28, 22.30, 11.35. MALDI-TOF: C_19_H_22_NS^+^ calculated 296.147, found 296.495.

### Spectrophotometric Measurements

Stock solutions of sodium or potassium salts of anions (10 mM) were prepared in water. ZM-FES stock solutions (1.0 mM) were prepared in ACN and subsequently diluted to a working concentration of 10 µM in ACN/H_2_O (1:1, v/v). For UV-Vis absorption and fluorescence emission studies, solution volumes were maintained at 1.0 mL for UV-Vis experiments and 3.0 mL for fluorescence experiments.

The absorption spectra of ZM-FES, both in the absence and presence of anion species, were measured over the wavelength range of 200–600 nm. Fluorescence emission spectra were collected between 400 nm and 730 nm using an excitation wavelength of 375 nm, with both excitation and emission slit widths set to 5 nm. Spectral readings were taken 5 min after the addition of each selected anion to the sensor solutions.

### Validation and Real Sample Applications

Furthermore, the fluorescence studies were also performed after spiking different concentrations (1, 3, 5 and 10 µM) of CN^−^ in ACN/H_2_O (1:1, v/v) to evaluate the accuracy of the method. To assess intra-day sensitivity, each concentration level was tested in parallel seven times; moreover, the entire procedure was repeated over three consecutive days to evaluate inter-day sensitivity.

The bitter almonds and the potatoes were obtained from a local market (Karaman province, Türkiye). The potatoes were stored in a moist soil environment until they began to sprout. The food extract samples were prepared following a previously reported method [44, 45]. The food samples (100 g) were initially crushed, followed by the addition of 50 mL of water and 1.0 g of NaOH. The mixture was then stirred for a duration of four days until the solution became turbid. The mixture was then transferred to a centrifuge tube and centrifuged at 7000 rpm for 5 min. The supernatant was filtered to obtain a clear solution and the pH was adjusted to 7.5. After adding different concentrations of CN^−^ (3 µM, 5 µM, and 10 µM) and the sensor (final concentration 5 µM) to these solutions, the fluorescence spectra were collected.

## Results and Discussions

### Rationale of Work

**Scheme 1:**
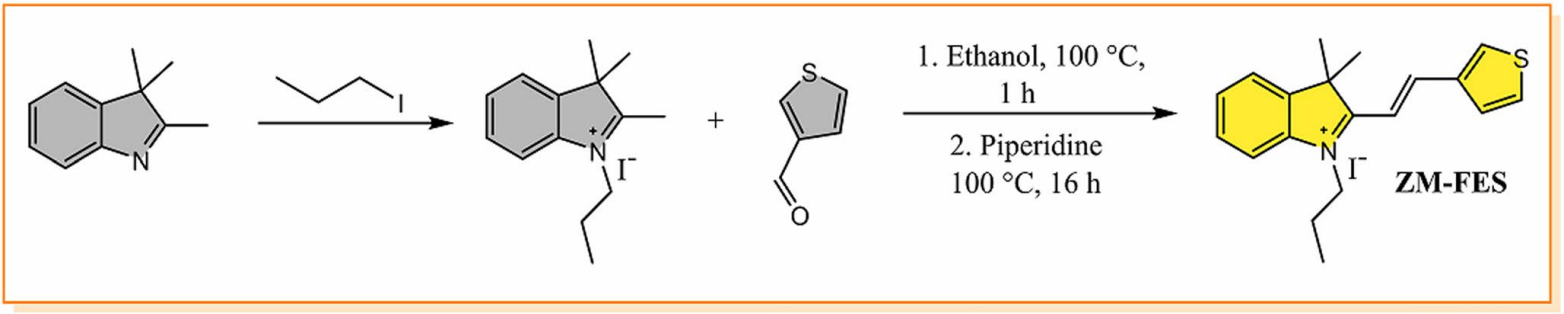
Synthesis of the sensor, ZM-FES

This research aims to develop a selective and sensitive sensor for the detection of cyanide ions in food samples (bitter almonds and sprouted potatoes) to meet the need for the protection of human health. One of the most effective and widely accepted approaches to the development of a cyanide probe is the use of indolium derivatives. Indolium and its derivatives have inspired the development of real-time detection sensors with high fluorescence quantum yield, low toxicity, large Stokes shift, excellent photostability and good biocompatibility. Moreover, since they employ a chemodosimetric approach, indolium-based (hemicyanine-based) receptors show high sensitivity and selectivity to cyanide ions [41]. Taking these facts into account, the ZM-FES sensor was synthesized through a two-step reaction (Scheme 1) and characterized by NMR and mass spectrometry (Fig. S1-Fig. S3).

### Spectroscopic Studies of the Sensor, ZM-FES

The binding interaction of the sensor ZM-FES (10.0 µM) with various anions (10.0 µM), including CN^−^, SO_3_^2−^, Br^−^, I^−^, Cl^−^, HPO_4_^2−^, H_2_PO_4_^−^, NO_3_^−^, NO_2_^−^, ClO_4_^−^, ClO_3_^−^, AcO^−^, SO_4_^2−^, CO_3_^2−^, HO^−^, and S^2−^ in ACN/H_2_O (1:1, v/v) mixture was examined through colorimetric analysis, as depicted in Fig. S4. The ZM-FES sensor initially synthesized as a deep yellow color, which changed to colorless upon the addition of CN⁻ ions. In contrast, the addition of other anions did not induce any color changes. This preliminary investigation indicates that the ZM-FES sensor can selectively detect cyanide ions, as evidenced by the distinct color variation.

**Fig. 1:**
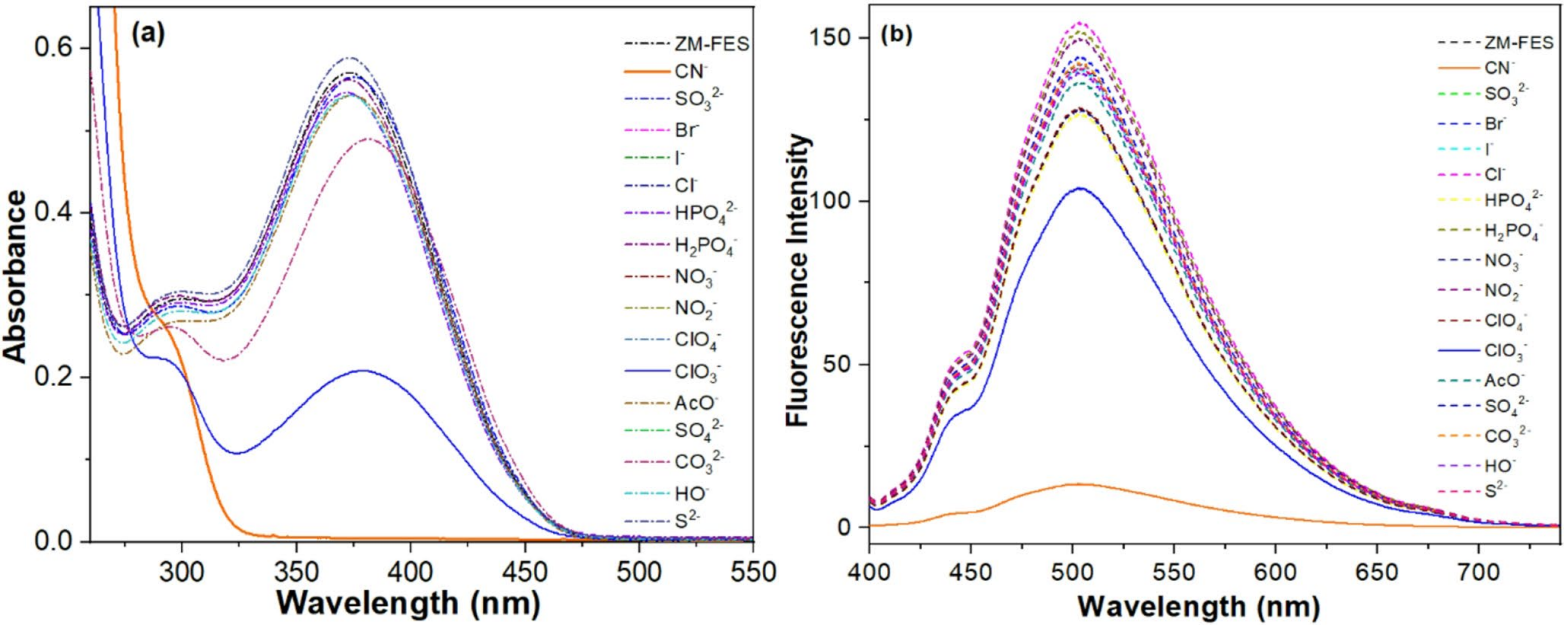
(**a**) Absorbance and (**b**) Fluorescence spectra of 10.0 µM ZM-FES to the anions tested (CN^−^, SO_3_^2−^, Br^−^, I^−^, Cl^−^, HPO_4_^2−^, H_2_PO_4_^−^, NO_3_^−^, NO_2_^−^, ClO_4_^−^, ClO_3_^−^, AcO^−^, SO_4_^2−^, CO_3_^2−^, HO^−^, and S^2−^) in ACN/H_2_O (1:1, v/v)

The detection of CN^−^ ions was additionally examined through absorption studies under conditions similar to those used in the naked-eye detection experiments. As can be seen from Fig. 1a, ZM-FES exhibited a band centered at 375 nm, which may be due to ICT across the sensor and this transition is responsible for the color of the probes [46, 47]. Furthermore, the addition of cyanide ion caused the complete disappearance of the band at 375 nm, whereas other added anions did not cause any change in absorbance. Only ClO_3_^−^ caused a slight decrease in the absorbance of ZM-FES.

The selectivity of ZM-FES was also investigated by fluorescence spectroscopy. To determine the fluorescence spectral response of ZM-FES to different anions, 1.0 equivalent of different anions (CN^−^, SO_3_^2−^, Br^−^, I^−^, Cl^−^, HPO_4_^2−^, H_2_PO_4_^−^, NO_3_^−^, NO_2_^−^, ClO_4_^−^, ClO_3_^−^, AcO^−^, SO_4_^2−^, CO_3_^2−^, HO^−^, and S^2−^) were added separately to the sensor ZM-FES solution (10 µM) and then the emission spectra were recorded in ACN/H_2_O (1:1, v/v) medium under λex = 375 nm. As depicted in Fig. 1b, the fluorescence spectrum of ZM-FES displayed a strong emission band at 502 nm in ACN/H_2_O (1:1, v/v) mixture. Although adding one equivalent of the tested anions did not result in substantial modifications to the fluorescence emission spectra of ZM-FES, the presence of cyanide ions (CN^−^) caused substantial quenching in fluorescence intensity, reaching a value of – 91.6%.

**Fig. 2:**
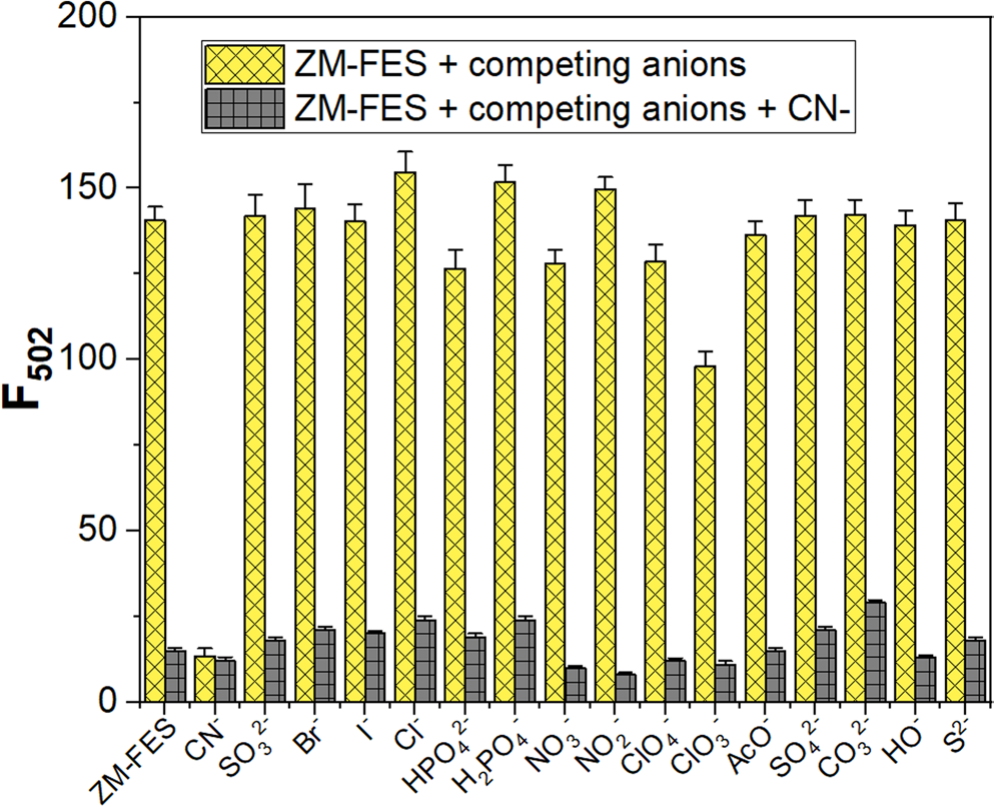
Fluorescence responses of 10.0 µM ZM-FES to the presence of 20.0 µM anions tested (yellow bars) and the subsequent addition of 10.0 µM CN^−^ (gray bars) in ACN/H_2_O (1:1, v/v). (Excitation wavelength = 375 nm)

The cyanide-sensing performance of the sensor was evaluated when exposed to various competitive anions. In this investigation, the fluorescence emission and absorbance spectrum of the sensor ZM-FES (10 µM) were first recorded in the presence of different anions (20 µM) (yellow bars in Fig. 2 and Fig. S5). The fluorescence emission and absorbance spectra of the same samples were then measured after the addition of cyanide ion (10 µM) (gray bars in Fig. 2 and Fig. S5). The results showed a decrease in fluorescence and absorbance intensity with the addition of cyanide ions, both for the ZM-FES sensor alone and for the ZM-FES sensor in combination with other anions. These results indicate that ZM-FES has high selectivity for cyanide ions and is minimally affected by other competing anions.

Furthermore, an investigation was conducted to examine the effect of pH on CN^−^ detection with ZM-FES, monitored through absorption analysis. The absorption spectra of ZM-FES, both in the presence and absence of CN⁻, were recorded across a pH range of 2.1 to 9.3 (Fig. S6). In the absence of CN⁻, the sensor showed no significant response to hydrogen ions between pH 2.1 and 7.1, with only minimal changes in absorption intensity, while a noticeable increase in absorption intensity was observed between pH 8.2 and 9.3. However, since the addition of CN⁻ leads to a pronounced decrease in absorbance (at 375 nm), this intrinsic increase in absorption at higher pH values (8.2–9.3) is not of significant concern for sensing performance. In the presence of CN⁻, the absorbance intensity decreased dramatically and remained stable across the entire pH range of 2.1 to 9.3. These results indicate that ZM-FES can be effectively used for CN⁻ detection within the pH range of 2.1–9.3.

Given the necessity of a rapid detection of the guest anion by an effective sensor, a kinetics study was conducted to evaluate the reaction time of the sensor with CN^−^. The fluorescence response timescale was examined at various time intervals of 0, 1, 2, 5, 10, 15, 20, 25, 30, 40, 50, 70, and 90 min (Fig. S7). The fluorescence intensity of the ZM-FES solution underwent a gradual decline, as CN^−^ was added ultimately achieving equilibrium within 5 min. Due to these results, all spectra in this study were obtained 5 min following the addition of CN^−^ and other anions in this study. Additionally, as demonstrated in Fig. S7, ZM-FES + CN^−^ exhibited stability in ACN/H_2_O (1:1, v/v) for a minimum of 90 min.

The selectivity of ZM-FES was also investigated with various metal ions such as K^+^, Na^+^, Ca^2+^, Al^3+^, Mg^2+^, Ni^2+^, Zn^2+^, Hg^2+^, Cd^2+^, Pb^2+^, Co^2+^, Cu^2+^, Mn^2+^, Cr^3+^, Ag^+^ and Fe^3+^ using UV-Vis spectroscopy. In metal ion selectivity studies of the ZM-FES sensor, it was found that ZM-FES showed no selectivity towards any of the metal ions tested (Fig. S8).

### Binding Studies

**Fig. 3:**
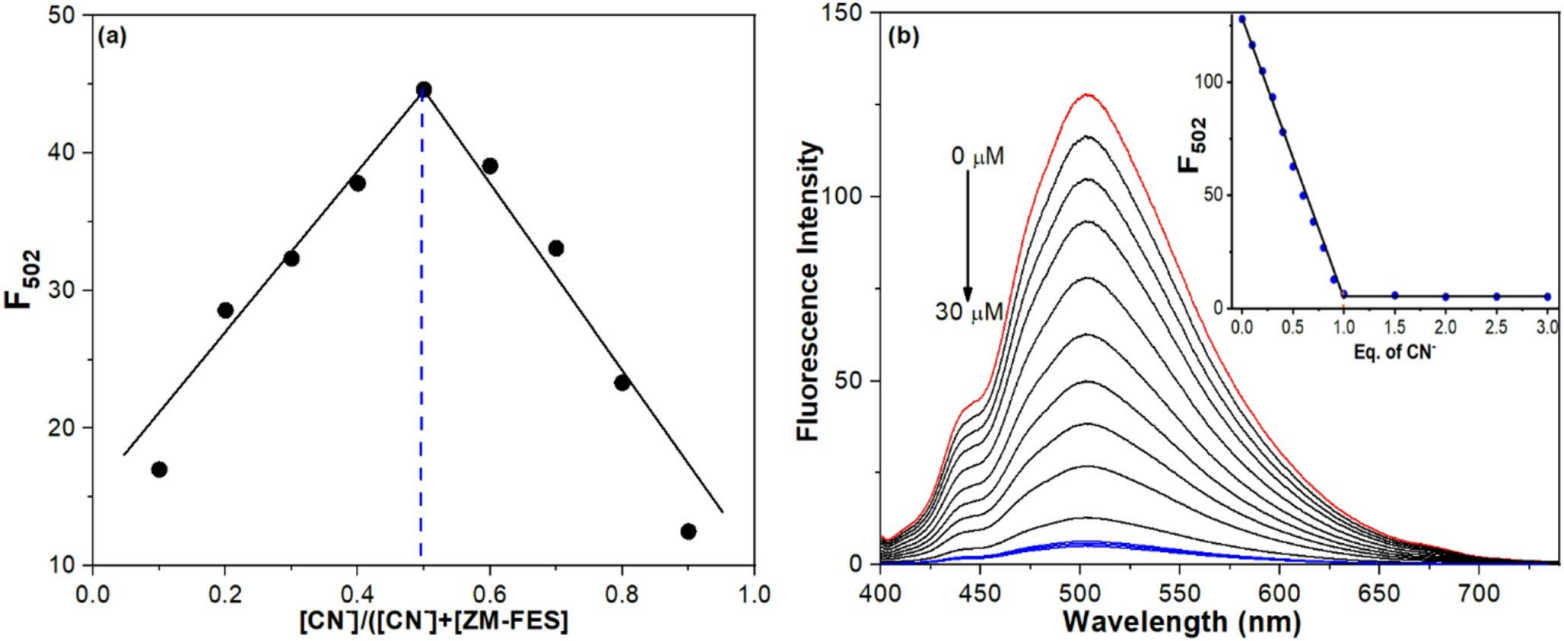
(**a**) Job’s plot for the interaction between ZM-FES and CN^−^. (**b**) Fluorescence titration of 10.0 µM ZM-FES with increasing concentrations (0, 1.0, 2.0, 3.0, 4.0, 5.0, 6.0, 7.0, 8.0, 9.0, 10.0, 15.0, 20.0, 25.0 and 30.0 µM, respectively) in ACN/H_2_O (1:1, v/v) of CN^−^

A detailed investigation was conducted to ascertain the binding mode between ZM-FES and CN^−^. This study employed Job’s plot, fluorescence titration, absorption titration, and mass spectroscopy to obtain insights into the binding interaction. The stoichiometry between ZM-FES and CN^−^ was first confirmed by Job’s method [48]. When the molar fraction of ZM-FES reached approximately 0.5, the fluorescence value changes at 502 nm approached a maximum (Fig. 3a), indicating a 1:1 reaction mode between compound ZM-FES and CN^−^. Upon examining the absorption titration results of ZM-FES with CN^−^, as shown in Fig. S9, the absorption intensity of ZM-FES (30 µM) at 375 nm gradually decreased with the addition of varying concentrations of CN^−^, eventually reaching saturation upon the addition of 1.0 equivalent of CN^−^. This indicates a 1:1 stoichiometric relationship between ZM-FES and CN^−^. The 1:1 stoichiometry was further confirmed by fluorescence titration experiments. As illustrated in Fig. 3b, the titration curve, which plots fluorescence intensities against varying concentrations of CN^−^, exhibited a linear increase and then plateaued at a 1:1 ratio of ZM-FES to CN^−^, confirming a 1:1 stoichiometry.

MALDI-TOF mass spectrometry was also used to confirm the binding stoichiometry between CN^−^ and ZM-FES. The mass spectrum of the ZM-FES/CN^−^ complex (in ACN/H_2_O (1:1, v/v)) displays a prominent peak at m/z = 322.66 (Fig. S10), which corresponds to a 1:1 interaction [(ZM-FES + CN^−^)]⁺ (calculated m/z = 322.150).

**Fig. 4:**
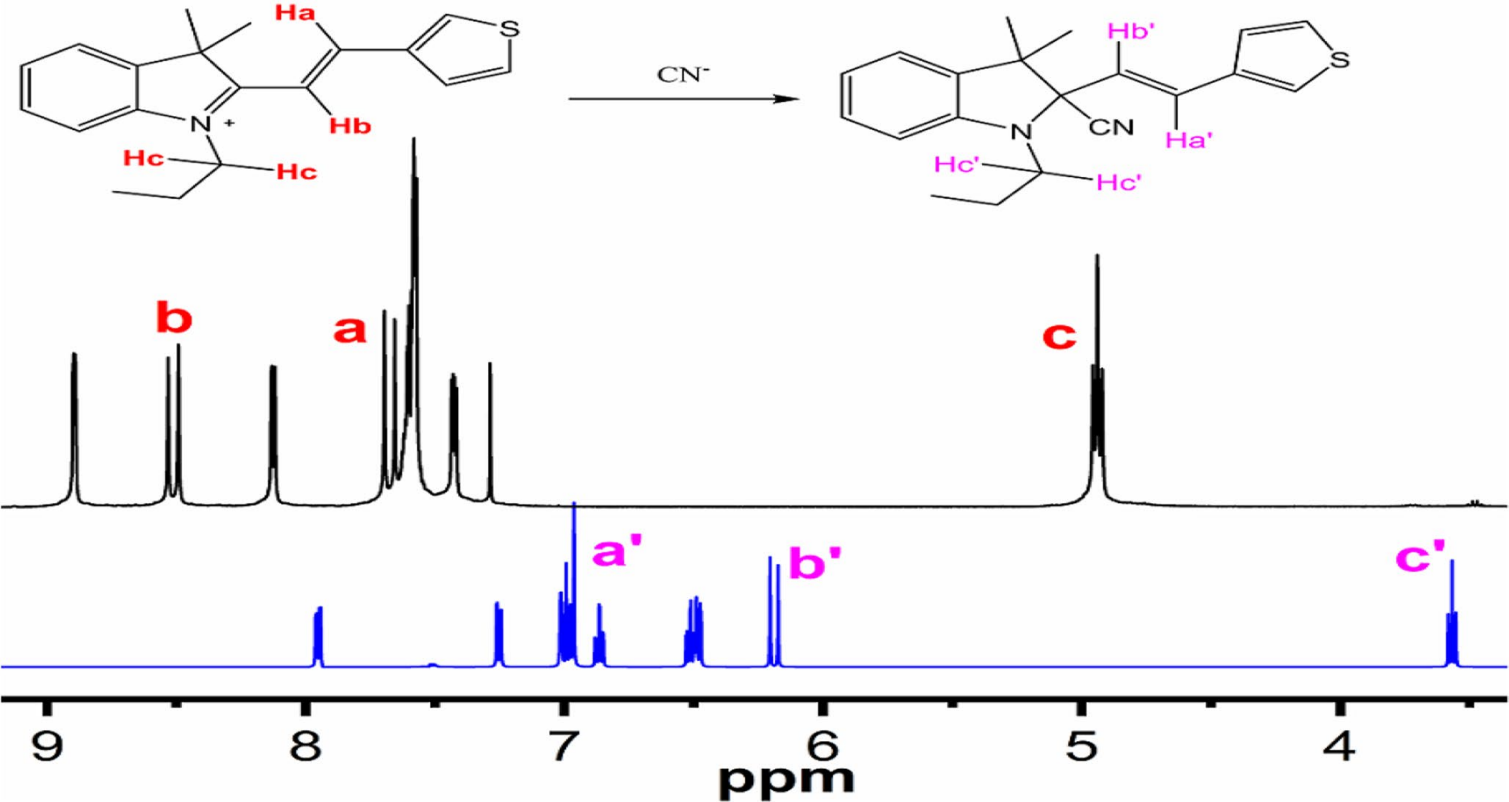
^1^H NMR spectra of ZM-FES (black line) and ZM-FES + CN^−^ (blue line)

Moreover, the ^1^H NMR titration of ZM-FES with CN^−^ in chloroform-d was examined. Following the addition of excess CN^−^ (tetrabutylammonium cyanide), a notable proton shift was observed. However, the reduced electron-withdrawing ability of the quaternary nitrogen atom of the indole is responsible for the olefinic proton’s upfield shift in the ZM-FES product. A shift in the position of the vinyl protons from δ 7.70 (Ha) and δ 8.53 (Hb) to δ 6.93 and δ 6.16, respectively, was also observed (Fig. 4). Furthermore, a consequence of the reduced electron density of the indole ring resulting from the quaternisation of N atom is that the N-CH_2_- protons of the ZM-FES are magnetically deshielded. After CN^−^ addition, these protons are upfield shifted (from δ 4.94 to δ 3.56).

**Scheme 2:**
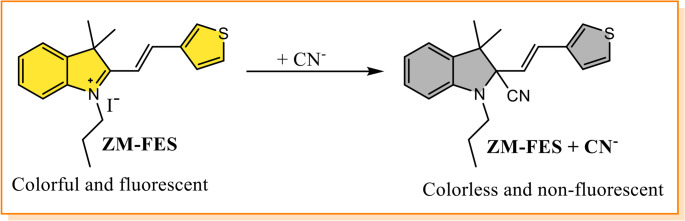
Proposed response mechanism between ZM-FES and CN^−^

Based on the data presented above, it can be concluded that ZM-FES interacts with CN^−^ in a 1:1 stoichiometric ratio and undergoes a nucleophilic addition reaction [41]. The proposed sensing mechanism between ZM-FES and CN^−^ is depicted in Scheme 2.

### Detection Limit Studies

**Fig. 5:**
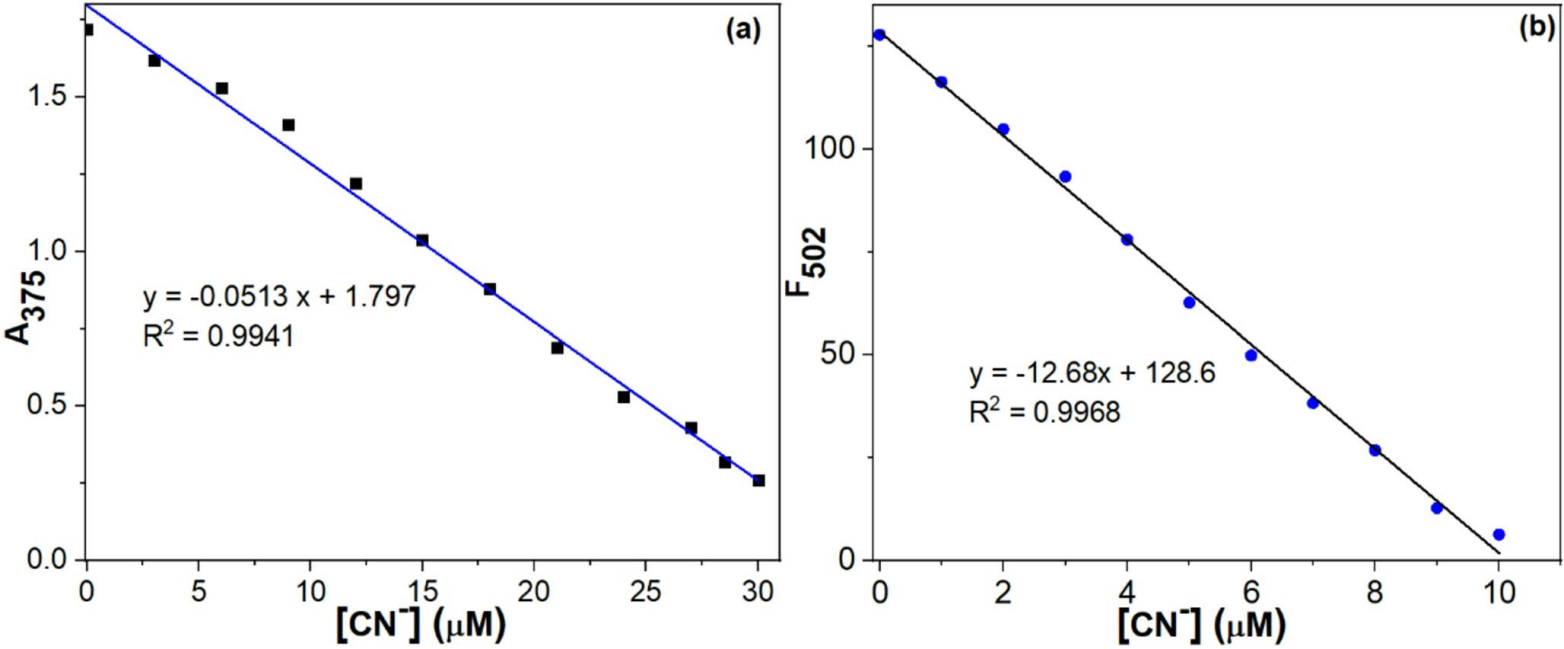
(**a**) Linear relationship between absorbance intensity and CN^−^ concentration (0.0–30.0 µM). Detection limit of ZM-FES for CN^−^ by UV-Vis method (Measurements were taken ACN/H_2_O (1:1, v/v)) (**b**) Linear relationship between fluorescence intensity and CN^−^ concentration (0.0–10.0 µM). Detection limit of ZM-FES for CN^−^ by fluorescence method (Measurements were taken ACN/H_2_O (1:1, v/v))

The limit of detection (LOD) for ZM-FES was calculated based on fluorescence and absorbance titrations. LOD is based on the formula 3 σ/k [49], where k is the regression equation’s slope and the blank measurements’ standard deviation. For this purpose, the absorbance or emission intensity of CN^−^ without ZM-FES was measured to ascertain the σ/k ratio. Ten subsequent measurements of blank samples were taken to calculate the standard deviation. As illustrated in Fig. 5a, the change in absorbance intensity was linearly related to the CN^−^ concentration within the 1–30 µM range (R² = 0.9941). The absorbance-based LOD was calculated to be 0.218 µM. Similarly, the fluorescence-based LOD was calculated [50]. Figure 5b demonstrates that fluorescence intensity is linearly correlated to the CN^−^ concentration in the range of 1–10 µM (R^2^ = 0.9968). The fluorescence-based LOD, as determined using the 3 σ/k calculation, is 0.195 µM. Both calculated LODs demonstrate that ZM-FES functions at levels significantly lower than the WHO standard for cyanide in drinking water (1.9 µM).

A comparison of ZM-FES with other reported cyanide-sensitive fluorescence sensors is shown in Table S1. The advantages of ZM-FES over other sensors include straightforward synthesis procedures, a low detection limit, a relatively fast response time, and practical applications.

### Validation and Real Sample Studies

Prior to the validation and real sample analyses, interference studies were conducted to evaluate potential matrix components that may be present in food samples. As shown in Fig. S11, the tested components did not affect the selectivity toward CN⁻.

The accuracy of the method was assessed by spiking 1, 3, 5 and 10 µM CN^−^ in ACN/H₂O (1:1, v/v) and analyzing the resulting fluorescence spectra. As shown in Table 1, the recovery rates (REC %) ranged from 95.0% to 103.7%. Furthermore, the intra-day precision and inter-day precision RSD (%) values remained at 3.0% and 7.9%, respectively, confirming excellent repeatability. Overall, the results showed that the ZM-FES sensor provides high accuracy in cyanide detection.

**Table 1:** Validation of CN^−^ detected with ZM-FES sensor in spiked samples

Samples	Added (µM)	Found (µM)	Recovery (REC %)	Precision RSD (%, *n* = 3)	
				Intra-day	Inter-day
1	1.0	0.95	95.0	1.3	2.8
2	3.0	3.0	100.1	2.4	3.6
3	5.0	4.9	97.9	1.6	5.5
4	10.0	10.4	103.7	3.0	7.9

Condition: [ZM-FES] = 10 µM in ACN/H_2_O (1:1, v/v)

A standard spike recovery test was conducted to assess the reliability of the developed ZM-FES sensor for the detection of CN^−^ in real samples. As shown in Table 2, the detected cyanide concentrations in bitter almond and sprouting potato extracts were found to be 1.69 µM and 1.27 µM, respectively. These values are similar to previously reported cyanide levels in comparable food samples, confirming the reliability of the method [51–52]. In addition, CN- recoveries were determined ranging from 82.52% to 96.92% and 85.25% to 96.63% for bitter almonds and sprouting potatoes, respectively, demonstrating the reliability and suitability of the probe for practical applications in detecting cyanide levels in food samples. The relative standard deviation (RSD %) of these measurements was less than 3% (*n* = 3). These findings suggest that ZM-FES is a precise and efficient instrument for cyanide detection, making it an important tool for food safety assurance.

**Table 2:** Quantitative detection of CN^−^ in bitter almond and sprouting potato extract samples

Sample	Detected (µM)	Added (µM)	Found (µM)	Recovery (%)	RSD (*n* = 3)
Bitter almond	1.69	3.0	3.87	82.52	1.99
		5.0	5.73	85.65	2.47
		10.0	11.33	96.92	1.86
Sprouting potato	1.27	3.0	3.64	85.25	1.12
		5.0	5.29	88.37	2.09
		10.0	10.89	96.63	1.89

Relative to other hemicyanine-sensitive fluorescence sensors documented in Table S1, ZM-FES presents multiple benefits such as simple synthesis methods, a minimal detection threshold, a comparatively quick response time, and practical applications.

## Conclusion

In this study, we developed a hemicyanine-based colorimetric turn-off fluorescent sensor, ZM-FES, capable of selectively detecting cyanide ions. ZM-FES was synthesized by combining an indolium moiety with a thiophene moiety, enabling high selectivity and sensitivity toward CN^−^. CN^−^ detection occurred via 1:1 stoichiometric nucleophilic addition to the indolium group, confirmed by Job’s plot, NMR, and MALDI-TOF. The sensor demonstrated a turn-off response to CN^−^ with minimal interference from other competing anions, along with remarkable stability over a wide pH range. ZM-FES successfully quantified CN^−^ in bitter almonds and sprouting potatoes, validating its utility in real food samples. The sensor’s rapid response times and simple preparation make it suitable for on-site food safety monitoring. Compared to conventional techniques (e.g., HPLC, GC-MS), ZM-FES offers cost-effectiveness, ease of use without requiring complex instrumentation.

## Supplementary Information

Below is the link to the electronic supplementary material.

Supplementary Material 1

## Data Availability

All data are provided in full in the results section of this paper and supplementary information accompanying this paper.
