# Supplementary material for: A Highly Selective Hemicyanine-Based Turn-Off Fluorescent Sensor for Cyanide Detection in Food Samples
Source: J Fluoresc. 2026 May 8;36(5):3553–62. doi: 10.1007/s10895-026-04788-3 (PMC13226347; doi:10.1007/s10895-026-04788-3)
Supplement: Supplementary file 1 — Supplementary Material 1 [file 10895_2026_4788_MOESM1_ESM.doc]

**A highly selective hemicyanine-based turn-off fluorescent sensor for cyanide detection in food samples**

Fuat Gokbel^a*^, Ziya Aydin^b^, Şeyma Akın^a^, Esma Nur Çenet^c^, Mustafa Keles^d^

^a^Karamanoğlu Mehmetbey University, Faculty of Engineering, Department of Food Engineering, 70200, Karaman, Türkiye

^b^Karamanoğlu Mehmetbey University, Vocational School of Technical Sciences, 70200 Karaman, Türkiye

^c^Karamanoğlu Mehmetbey University, Faculty of Engineering, Department of Bioengineering, 70200, Karaman, Türkiye

^d^Osmaniye Korkut Ata University, Faculty of Arts and Sciences, Department of Chemistry, 80000, Osmaniye, Türkiye

^*^Corresponding Author. Email: [fgkbel@kmu.edu.tr](mailto:fgkbel@kmu.edu.tr), [fgkbel@hotmail.com](mailto:fgkbel@hotmail.com)


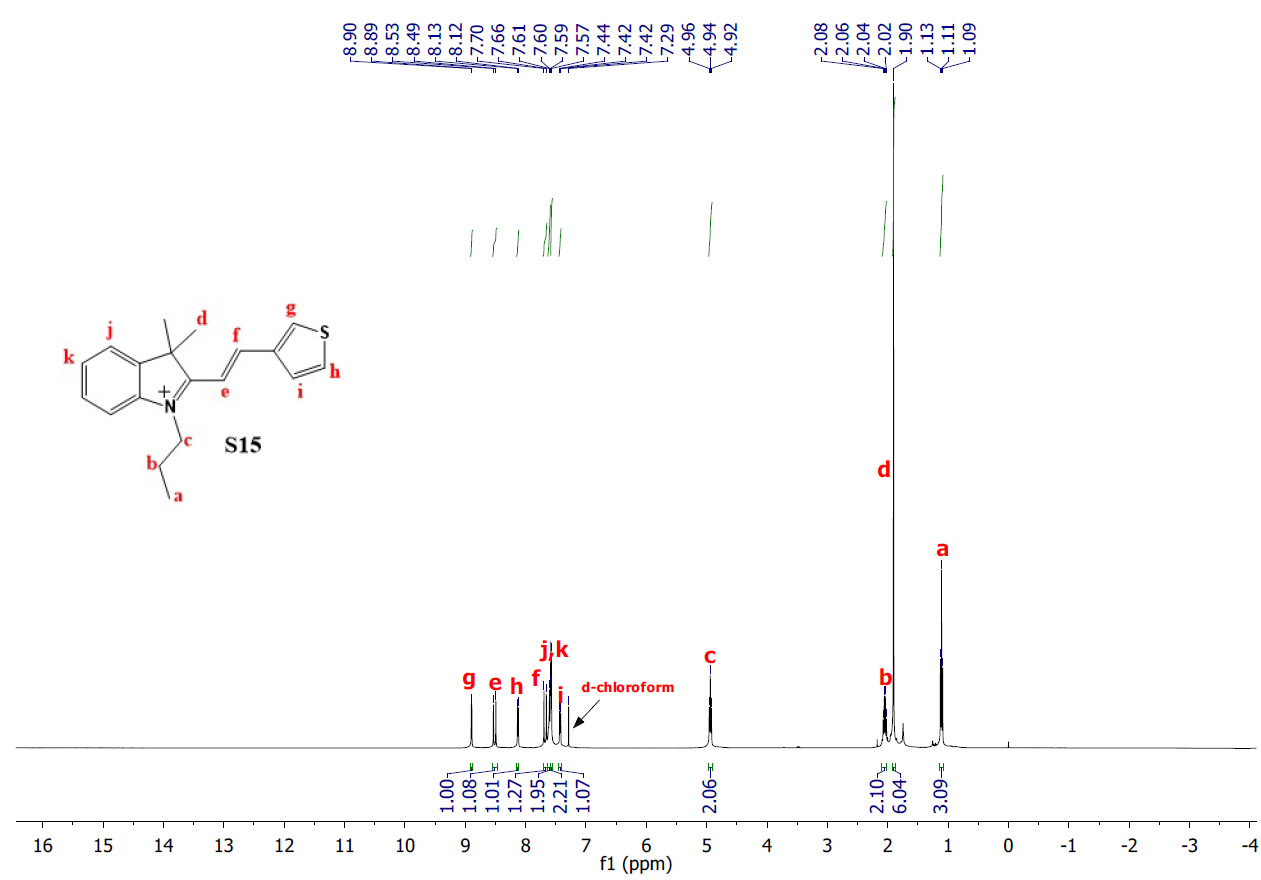


**Figure S1.** ^1^H NMR spectrum of ZM-FES.


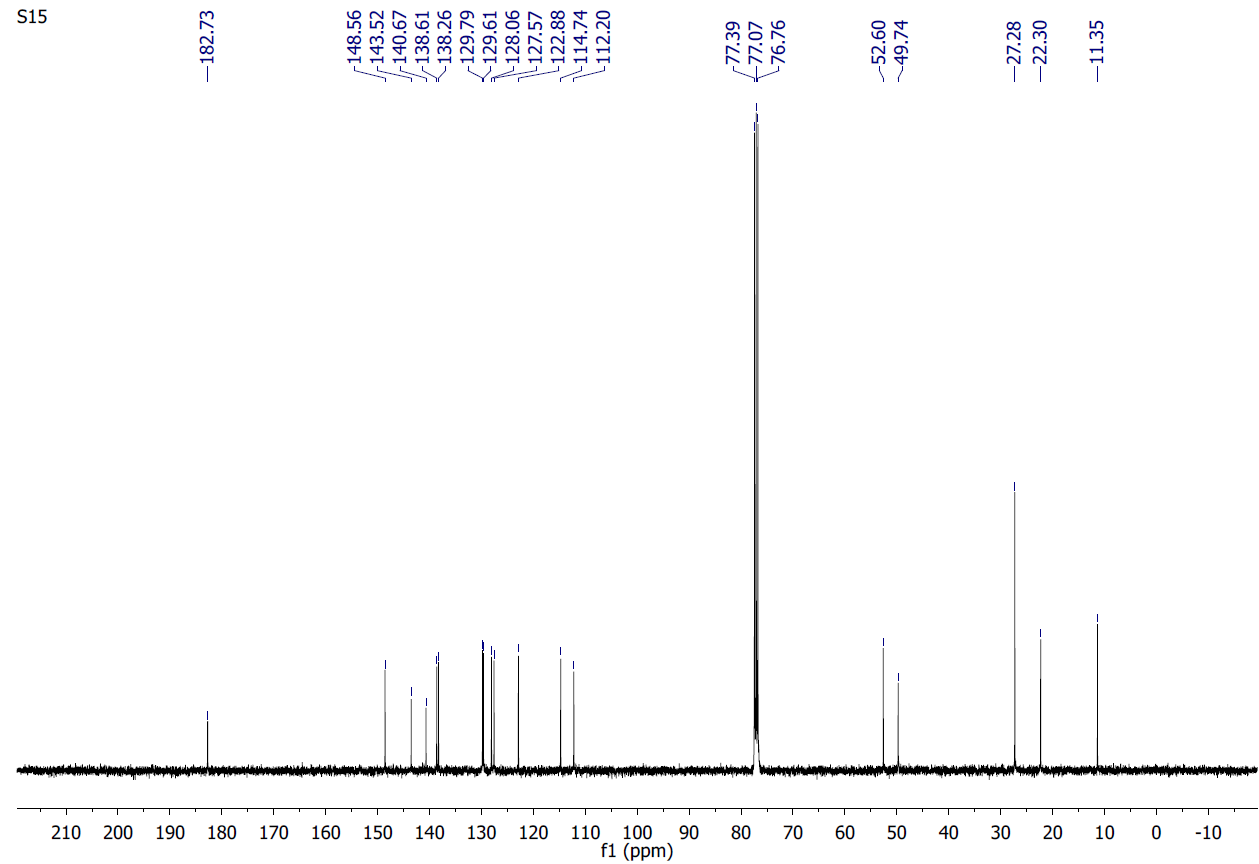


**Figure S2**. ^13^C NMR spectrum of ZM-FES.


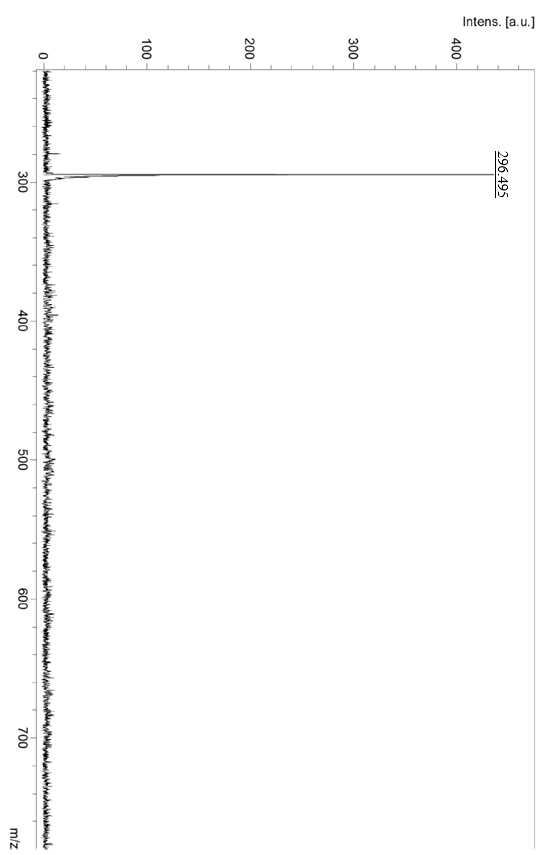


**Figure S3.** MALDI-TOF spectrum of ZM-FES.


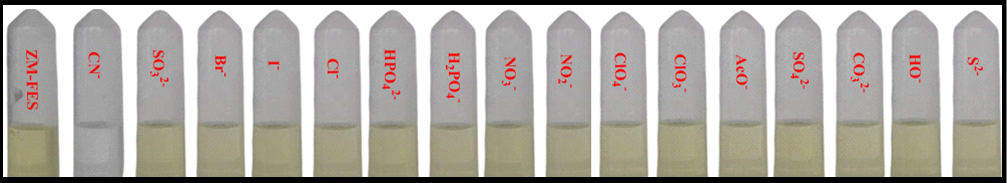


**Figure S4.** Color changes of 10 μM ZM-FES with various anions (20 μM for CN^-^, SO_3_^2-^, Br^-^, I^-^, Cl^-^, HPO_4_^2-^, H_2_PO_4_^-^, NO_3_^-^, NO_2_^-^, ClO_4_^-^, ClO_3_^-^, AcO^-^, SO_4_^2-^, CO_3_^2-^, HO^-^, and S^2-^) in ACN/H_2_O (1:1, v/v).


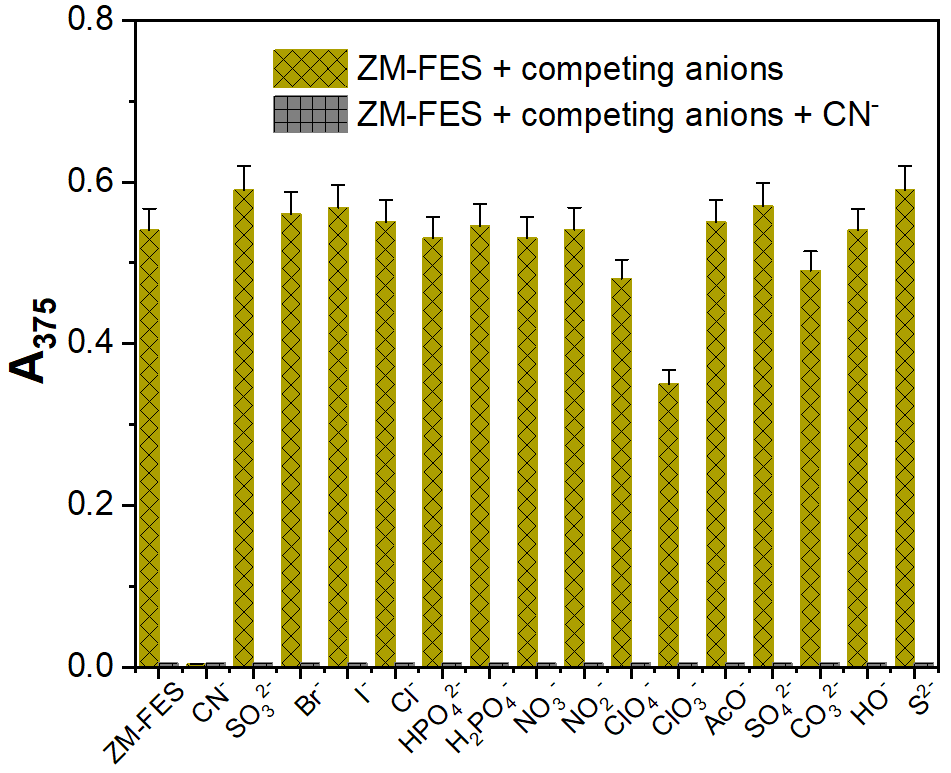


**Figure S5.** Absorbance responses of 10.0 µM ZM-FES to the presence of 20.0 µM anions tested (yellow bars) and the subsequent addition of CN^-^ (gray bars) in ACN/H_2_O (1:1, v/v).


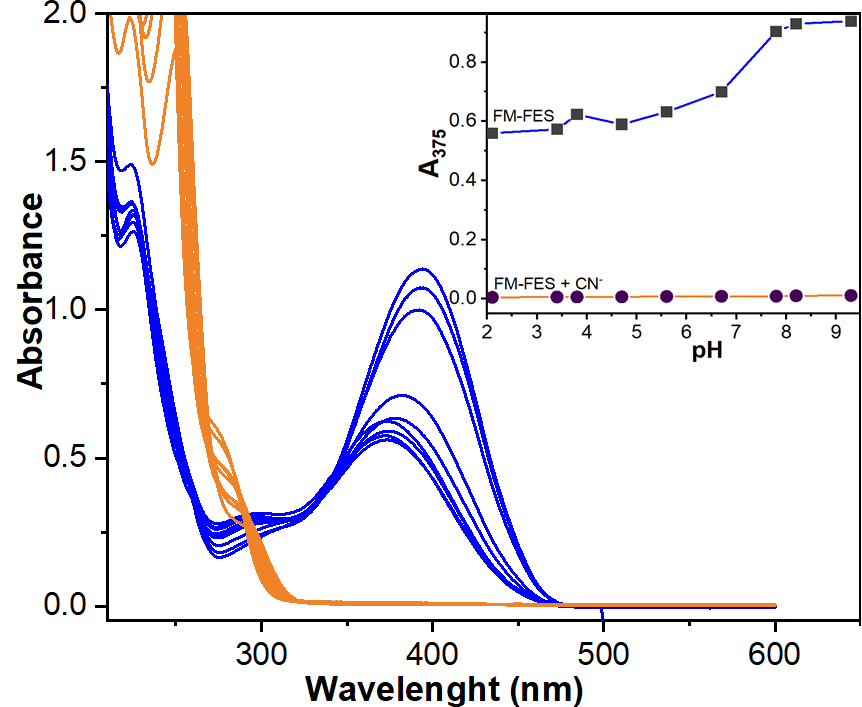


**Figure S6.** Effect of pH on the absorbance intensity of ZM-FES (20 µM) and ZM-FES+CN^-^ (20 µM) at various pH values ACN/Buffered solution (1:1, v/v).

**
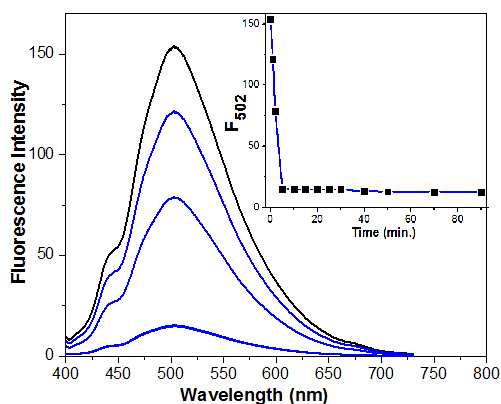
**

**Figure S7.** Fluorescence spectra of ZM-FES+CN^-^ (10.0 µM) collected at different reaction time intervals (0, 1, 2, 5, 10, 15, 20, 25, 30, 40, 50, 70, and 90 min) in ACN/H_2_O (1:1, v/v).


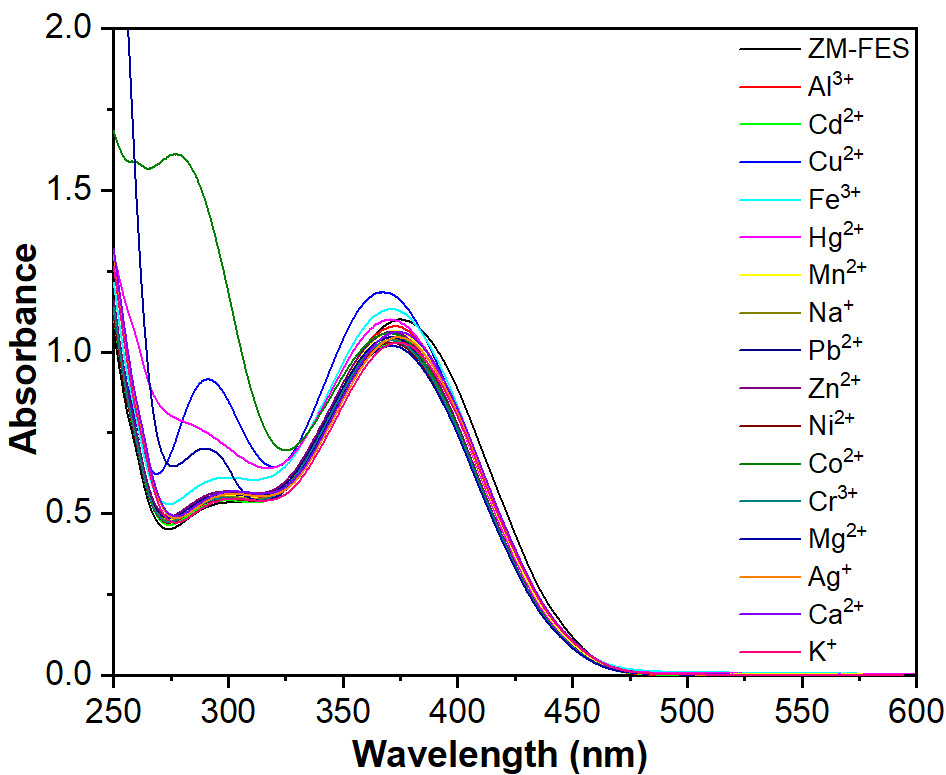


**Figure S8**. The absorbance spectra of the sensor (20 µM) with various metal ions (40 µM) in ACN/H_2_O (1:1, v/v).


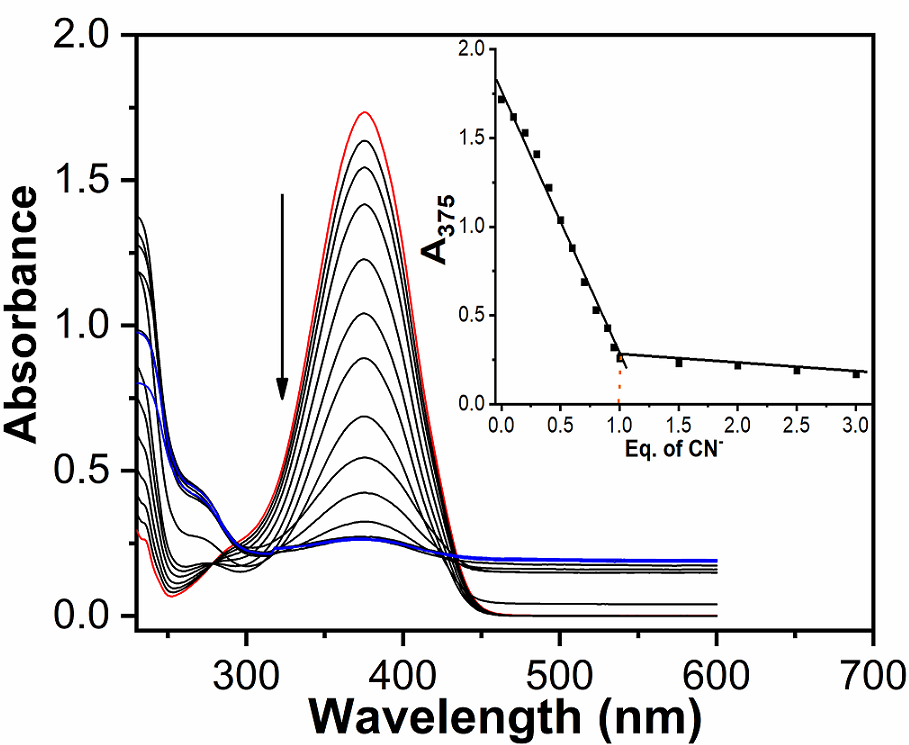


**Figure S9.** Absorbance titration of 30.0 µM ZM-FES with increasing concentrations (0, 3.0, 6.0, 9.0, 12.0, 15.0, 18.0, 21.0, 24.0, 27.0, 30.0, 45.0, 60.0, 75.0 and 90.0 µM, respectively) in ACN/H_2_O (1:1, v/v) of CN^-^.


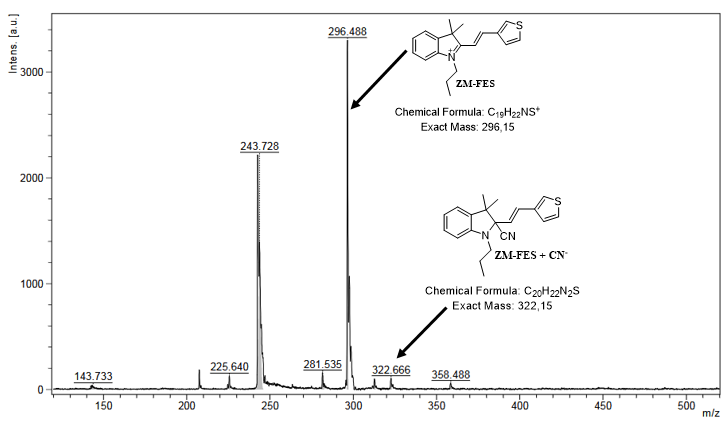


**Figure S10.** MALDI-Tof Mass Spectrum of ZM-FES+CN^-^


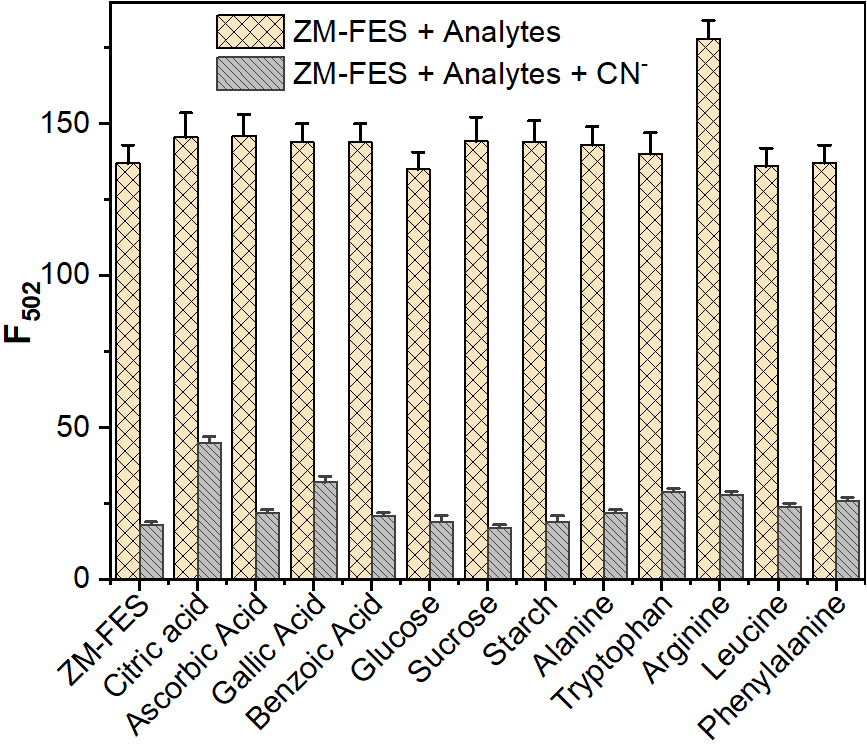


**Figure S11.** Bar chart illustrating the fluorescence intensity at 502 nm for ZM-FES in the presence of potentially interfering analytes (yellow bars) and after the subsequent addition of CN^-^ (gray bars).

**Table S1.**Comparison of several reported hemicyanine-based sensors for the detection of CN-

| Structure/Material | Testing Media | Response time | Interference | Detection limit | Ref. |
| --- | --- | --- | --- | --- | --- |
|  | DMF/H_2_O | < 3 min. | None | 0.018 µM | [S1] |
|  | DMSO/H_2_O  (1:1, v/v) | NA | None | NA | [S2] |
|  | THF | < 1 min. | None | 0.021 µM | [S3] |
|  | DMSO-buffer (Tris–HCl, 10.0 mM, pH ¼7.4,6:4, v/v) | 10 min. | None | 0.408 µM | [S4] |
|  | DMSO/ H_2_O  (1:9, v/v) | NA | None | 0.14 µM | [S5] |
|  | H_2_O | NA | None | 0.334 µM | [S6] |
|  | ACN | < 2 min. | None | 0.7 µM | [S7] |
|  | DMF/H_2_O  (2:8, v/v) | NA | None | 17.3 µM | [S8] |
|  | 50% aq DMSO | < 1 min. | HS^-^ | 0.43 µM | [S9] |
|  | ACN/H_2_O  (1:1, v/v) | 5 min. | None | 0.218 µM (for Absorption studies)  0.195 µM (for Fluorescence studies) | This study |

NA: not available

**References**

[S1] Sun, Y., Fan, S., Duan, L., Li, R. (2013). A ratiometric fluorescent probe based on benzo [e] indolium for cyanide ion in water. *Sensors and Actuators B: Chemical*, *185*, 638-643.

[S2] Gwon, S. Y., Lee, E. M., Kim, S. H. (2012). Hemicyanine-based colorimetric chemosensors: Different recognition mechanisms for CN− sensing. Spectrochimica Acta Part A: Molecular and Biomolecular Spectroscopy, 96, 77-81.

[S3] Yue, Y., Huo, F., Yin, C., Chao, J., Zhang, Y. (2015). A new “donor-two-acceptor” red emission fluorescent probe for highly selective and sensitive detection of cyanide in living cells. Sensors and Actuators B: Chemical, 212, 451-456**.**

[S4] Wang, S., Fei, X., Guo, J., Yang, Q., Li, Y., Song, Y. (2016). A novel reaction-based colorimetric and ratiometric fluorescent sensor for cyanide anion with a large emission shift and high selectivity. Talanta, 148, 229-236.

[S5] Palanisamy, J., Gatasheh, M. K., Hatamleh, A. A. (2024). A reaction based carbazole–indolium conjugate probe for the selective detection of environmentally toxic ions. Analytical Methods, 16(18), 2869-2877.

[S6] Li, J., Chang, Z., Pan, X., Dong, W., & Jia, A. Q. (2019). A novel colorimetric and fluorescent probe based on indolium salt for detection of cyanide in 100% aqueous solution. Dyes and Pigments, 168, 175-179.

[S7] Lyngkhoi, D. L., Khatua, S. (2024). A coumarin containing hemicyanine-based probe for dual channel detection of cyanide ion. Inorganica Chimica Acta, 572, 122300.

[S8] Gosi, M., Marepu, N., Sunandamma, Y. (2021). Cyanine-based fluorescent probe for cyanide ion detection. Journal of Fluorescence, 31(5), 1409-1415.

[S9] Magesh, K., Vijay, N., Wu, S. P., Velmathi, S. (2023). Dual-responsive benzo-hemicyanine-based fluorescent probe for detection of cyanide and hydrogen sulfide: Real-time application in identification of food spoilage. Journal of Agricultural and Food Chemistry, 71(2), 1190-1200.
